# 95 Moral Injury and Four-Quadrant Approach to Ethics Issues in Burn Care

**DOI:** 10.1093/jbcr/irae036.094

**Published:** 2024-04-17

**Authors:** Eva Regel

**Affiliations:** Massachusetts General Hospital, Westborough, Massachusetts

## Abstract

**Introduction:**

The presentation will address the post-pandemic changes that take place in current healthcare in general and burn care specifically, such as dramatically increased rates of burnout and staff turnover. Particular attention will be given to introducing and discussing the concept of moral injury and its relevance to the everyday work in the Burn unit; the presentation will address and discuss the impact and causes of moral injury on nursing staff and clinicians in the context-specific Burn care setting. Particular attention will be devoted to the effects of moral injury on the paradigm of the “four-quadrant approach” commonly used in ethical decision-making in burn care. The presentation will offer a discussion of the moral underpinnings of moral injury and moral responsibility and the relevance of these complexities on providers’ ability to employ the “four-quadrant approach " will be discussed. The current research on moral injury and distress will be highlighted in the presentation.

**Methods:**

Discussion

**Results:**

Results will be based on a literature review.

**Conclusions:**

Several possible solutions to mitigate the effects of moral injury will be offered. Specifically, changes to nursing curriculums and medical schools will be proposed.

**Applicability of Research to Practice:**

Direct application as recognition and mitigation of the effects of moral injury can have a direct effect on decision making process as well as sustainability and resilience of staff on the Burn unit.

